# Sex-specific assessment of reduced coronary sinus flow in non-hypertensive patients with coronary artery disease at rest

**DOI:** 10.3402/ljm.v8i0.21553

**Published:** 2013-07-16

**Authors:** Xiao-Zhi Zheng, Bin Yang, Jing Wu

**Affiliations:** 1 Department of Ultrasound, Jinling Hospital, Nanjing University School of Medicine, Nanjing City, Jiangsu Province, People's Republic of China; 2 Department of Ultrasound, The Fourth Affiliated Hospital of Nantong University, The First People's Hospital of Yancheng, Jiangsu Province, People's Republic of China

**Keywords:** coronary flow, coronary sinus, coronary artery stenoses, males and females, non-hypertensive patients, transthoracic Doppler echocardiography

## Abstract

**Background:**

Access to data on the coronary flow in the coronary sinus (CS) can aid in the diagnosis of coronary artery disease (CAD). We tested the hypothesis that assessing the CS flow by transthoracic Doppler echocardiography (TTE) at rest can detect coronary artery stenosis in non-hypertensive patients.

**Methods:**

The antegrade phase of coronary flow in the CS was analyzed and compared in 140 male and 135 female non-hypertensive subjects who had all undergone coronary angiography.

**Results:**

There were statistically significant differences noted between males and females for the CS flow both in normal subjects and patients with CAD. Compared with normal subjects, patients with CAD had significantly lower blood flow in the CS both in males (196.6±174.31 vs. 367.65±168.04 ml/min, *P*<0.01) and females (183.04±65.46 vs. 244.13±135.43 ml/min *P*<0.01). For males, the diagnostic sensitivity, specificity, and accuracy of the cutoff value of the CS flow (206 ml/min) for predicting a significant coronary artery stenosis (>70%) were 91.67%, 81.25%, and 85.71%, respectively. For females, those of the cutoff value of the CS flow (195 ml/min) were 85.71%, 75%, and 80%, respectively.

**Conclusion:**

TTE can effectively detect coronary hemodynamically significant stenosis in non-hypertensive male and female patients at different cutoff values.

Coronary angiography is constantly considered as gold standard for the diagnosis of coronary artery disease (CAD). However, this method is expensive and requires invasive intervention and it cannot be performed in all patients easily. Due to its non-invasive and easily accessible nature, echocardiography has been used as a routine examination for assessing myocardial ischemia over the past decades. The conventional two-dimensional ultrasound and a variety of quantitative analyses of myocardial motion, such as color kinesis, myocardial velocity, displacement, strain, strain rate, and myocardial speckle imaging, all have difficulties in the early detection of myocardial ischemia at rest (1, 2).

The coronary sinus (CS) is the major venous collection system for the coronaries, especially for the left coronary artery (3, 4). Over the last decade, coronary flow reserve (CFR) in the CS has been used to diagnose significant coronary artery stenosis by transesophageal Doppler echocardiography (TEE) due to an inadequate visualization of the coronary arteries, especially their mid- and distal parts (5–7). TEE is a semi-invasive procedure and vasodilator drugs, such as adenosine, are not always harmless. In patients with CAD, they can induce angina pectoris and even more serious results. In our previous study, we have confirmed that reduced antegrade flow predicts coronary artery stenosis in hypertensive patients (8). As there is a difference in CS flow between the hypertensive and non-hypertensive patients due to a different heart work load (afterload) and a different blood pressure (diastolic pressure), whether transthoracic Doppler echocardiography (TTE) can detect hemodynamically significant stenosis of the coronary artery in non-hypertensive patients remains unknown.

The purpose of our study was to determine the role of the CS flow assessed by TTE in the diagnosis of severe coronary artery stenosis in non-hypertensive male and female patients.

## Materials and methods

### Study population

Our study was approved by the local human research ethics committee of The Fourth Affiliated Hospital of Nantong University (No. 2012000135), and informed consent was obtained from all of the subjects. The study population consisted of 275 non-hypertensive participants who had undergone coronary angiography because of suspected CAD: males: 80 normal subjects (mean age: 57.23±9.4 years, range: 48–67 years), 60 patients with CAD (mean age: 57.4±10.9 years, range: 46–68 years) with angiographically proven >50% stenosis; females: 63 normal subjects (mean age: 61.3±8.2 years, range: 53–70 years), 72 patients with CAD (mean age: 62.1±10.9 years, range: 52–72 years) with angiographically proven >50% stenosis. All of the subjects had a sinus rhythm, normal systolic function of the left and right ventricles, normal right ventricular systolic pressure, normal right atrial pressure, normal pulmonary artery pressure, and mitral and tricuspid valvular regurgitation less than grade 2. The subjects with congenital heart disease (*n*=1), valvular disease (*n*=3), cardiomyopathy (*n*=1), diabetes mellitus (*n*=2), pulmonary hypertension (*n*=1), intracoronary shunts (*n*=1), anomalous pulmonary venous return (*n*=1), and persistent left superior vena cava (*n*=1) were excluded from the study.

### Echocardiographic measurements

**Fig. 1:**
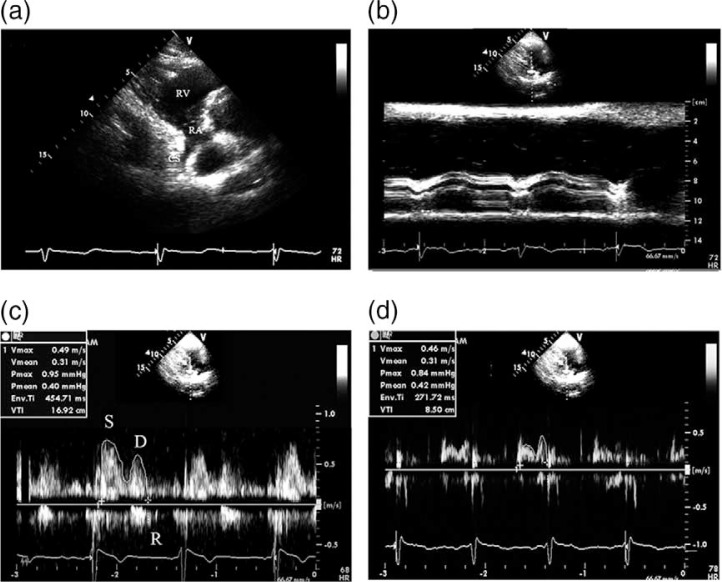
The measurement of coronary blood flow in the CS. (a) The CS seen from the parasternal right ventricular inflow tract view. (b) The measurement of the diameter of CS using adjust M-Mode ultrasonography. (c) The Doppler spectrum of coronary blood flow in the CS obtained from the parasternal right ventricular inflow tract view and the measurement of Doppler parameters of coronary blood flow in the CS by digitized Doppler spectral envelopes in normal subjects. (d) The measurement of Doppler parameters of coronary blood flow in the CS by digitized Doppler spectral envelopes in patients with CAD (d). RA = right atrium; RV = right ventricle; CS = coronary sinus; S = systolic wave of the antegrade blood flow; D = diastolic wave of the antegrade blood flow; R = retrograde blood flow; Env.Ti = duration of measured envelope; CAD = coronary artery disease.

The echocardiographic study was performed with the subject lying in a left lateral decubitus position. The echocardiographic data were acquired using a commercially available ultrasonic system (Vivid E9; General Electric Medical Systems, Milwaukee, WI, USA, equipped with an M5S single-crystal matrix-array transducer) as described previously (5, 9). First, the standard parasternal long axis view was obtained. An M-mode tracing (66.7 mm/s) was recorded for the following measurements: diastolic interventricular septal thickness (DIVST), diastolic posterior wall thickness (DPWT), left ventricular end-diastolic diameter (LVEDD), and left ventricular end-systolic diameter (LVESD). The left ventricular ejection-fraction (LVEF) was automatically calculated by the ultrasonic system. Second, the parasternal right ventricular inflow tract view was obtained. The transducer was manipulated to visualize the mouth of the CS (Fig. 1a). To avoid the influence of atrial contraction, the diameter of coronary sinus (DCS) was measured at a 1-cm distance from the mouth in the end diastolic phase before the P wave on ECG using adjusted M-mode tracing (Fig. 1b). A pulsed-wave sample volume (3 mm) was placed at a 1-cm distance from the mouth, and then rotated by a small amount (Doppler angle between the ultrasound beam and vessels <30°) to obtain the optimum Doppler flow signals, and spectral recordings of the flow were made (Fig. 1c and d).

### Analysis of coronary blood flow in the CS

The antegrade phase of coronary flow in the CS moving into the right atrium was analyzed. The velocity time integral (VTI, cm) was determined through digitized Doppler spectral envelopes (Fig. 1c and d). The blood flow per stroke (ml/stroke) and the blood flow per min (ml/min) in the CS was calculated according to the following formulae: blood flow per stroke = π×*D* ^2^/4×VTI; blood flow per min (ml/min) = π×*D* ^2^/4×VTI×heart rate (HR), where *π* is the ratio of the circumference of a circle to its diameter, and *D* is the CS diameter (5). The average value of three spectral and planimetric envelopes was used.

### Coronary angiography

Coronary angiography was performed in all participants by the femoral approach within 24 h of TTE according to the standard method of Judkins (10) using the COROSKOP Plus angiographic complex (Siemens AG, Berlin, Germany) and standard catheters and conventional views. The number of coronary arteries with significant stenoses, the localization of stenosis, and the maximal percentages of stenoses were determined.

### Reproducibility

Intraobserver variability was assessed in 30 randomly selected participants by repeating the measurements on two occasions (3 days apart) under the same basal conditions. To test the interobserver variability, the measurements were performed on the same subject by a second blinded observer. Variability was calculated as the mean percentage error, derived as the difference between the two sets of measurements, divided by the mean of the observations.

### Statistical analysis

Data were expressed as the mean±SD. The differences between the two groups were tested using an unpaired two-tailed *t*-test. A receiver operating characteristic curve (ROC) analysis was used to evaluate and compare the performance of the CS parameters in discriminating between patients with or without CAD. The sensitivity, specificity, positive predictive value, negative predictive value, and accuracy of coronary flow as a predictor of significant coronary artery stenosis were measured in the traditional manner. A value of *P*<0.05 was considered statistically significant. All statistical analyses were performed with SPSS version 13 software for Windows (SPSS Inc., Chicago, IL, USA).

## Results

### Clinical and echocardiographic parameters

As shown in Table 1, age, HR, systolic blood pressure (SBP), diastolic blood pressure (DBP), DIVST, DPWT, LVEDD, and LVEF did not differ between the normal subjects and the patients, neither did they differ between the males and the females. Compared with normal subjects, the non-hypertensive patients with CAD had significantly higher PP. There were statistically significant differences between males and females for the CS flow. Compared with normal subjects, patients with CAD had significantly smaller CS diameter (4.84±1.22 vs. 6.21±1.14 mm for males, *P*<0.05; 4.43±0.62 vs. 5.38±1.53 mm for females, *P*<0.05) and lower blood flow in the CS both in males (2.83±1.39 vs. 4.92±2.23 ml/stroke, *P*<0.05; 196.6±174.31 vs. 367.65± 168.04 ml/min, *P*<0.01) and females (2.04±1.18 vs. 3.76±2.47 ml/stroke, *P*<0.05; 183.04±65.46 vs. 244.13±135.43 ml/min, *P*<0.01).

**Table 1:** Clinical and echocardiographic parameters of normal subjects and non-hypertensive patients with CAD

	Normal subjects		Patients with CAD	
		
Parameters	Male	Female	Male	Female
Clinical parameters				
Age (years)	57.2±9.4	61.3±8.2	57.4±10.9	62.1±10. 9
HR (beats/min)	74.51±11.84	70.65±9.82	72.33±10.87	69.17±9.25
SBP (mm Hg)	118±20.79	109.34±26.19	115.44±24.36	105.07±25.22
DBP (mm Hg)	75.21±6.66	68.37±8.45	73.75±8.89	70.76±7.42
PP (mm Hg)	41.23±4.69	42.84±5.29	48.75±8.53*	47.56±10.36*
Echocardiographic parameters				
DIVST (mm)	9.68±1.02	9.94±1.21	9.96±1.05	10.01±1.22
DPWT (mm)	9.62±1.15	9.76±0.98	9.83±1.01	9.82±1.09
LVEDD (mm)	45.21±5.44	44.28±4.15	45.36±6.62	44.62±5.55
LVEF (%)	68.78±5.49	67.39±9.42	66.47±4.99	65.97±6.87
DCS (cm)	6.21±1.14	5.38±1.53	4.84±1.22*	4.43±0.62*
VTI (cm)	16.11±6.02	14.98±1.56	14.33±4.73	12.23±1.56
Flow (ml/stroke)	4.92±2.23	3.76±2.47▵	2.83±1.39*	2.04±1.18*▵
Flow (ml/min)	367.65±168.04	244.13±135.43▵	196.6±174.31**	183.04±65.46**▵

* *P* <0.05

** *P* <0.01, unpaired *t* test, compared to the values of normal subjects of the same gender

▵ *P* <0.05, unpaired *t* test, compared to the values of the males of the same group. CAD = coronary artery disease; HR = heart rate; SBP = systolic blood pressure; DBP = diastolic blood pressure; PP = pulse pressure; DIVST = diastolic interventricular septal thickness; DPWT = diastolic posterior wall thickness; LVEDD = left ventricular end-diastolic diameter; DCS = the diameter of coronary sinus; VTI = velocity time integral; LVEF = left ventricular ejection fraction.

### Coronary angiographic findings

The culprit coronary arteries where the stenotic lesions were located are shown in Table 2. The mean percentage diameter stenosis in males and females was 87.74 ±16.54% and 79.74 ±18.44%, respectively.

**Table 2:** Coronary angiographic findings in patients with CAD

	Patients with CAD	
	
Portion	Male	Female
LAD		
Proximal	5	7
Middle	42	53
Distal	24	31
LCX		
Proximal	3	7
Middle	27	23
Distal	3	6
RCA		
Proximal	6	5
Middle	7	9
Distal	4	3
FDB	17	12

CAD = coronary artery disease; LAD = left anterior descending coronary artery; LCX = left circumflex artery; RCA = right coronary artery; FDB = the first diagonal branch.

### The impact of PCA, the left and right coronary artery lesions on the CS flow in males and females

As shown in Table 3, the CS flow was significantly less in the proximity of coronary lesions (PCA) group than in the left and right coronary artery lesions groups (*P*<0.05) both in males and females, and in the respective group, the CS flow was significantly more in males than in females (*P*<0.05). The CS flow was significantly correlated with the mean percent diameter stenosis in the PCA group and the left coronary artery lesions groups (*r*=0.86, *P*<0.01; *r*=0.74, *P*<0.01, respectively in males, and *r*=0.83, *P*<0.01, *r*=0.71, *P*<0.01, respectively in females). The CS flow did not show a significant correlation with the mean percent diameter stenosis (*r*=0.12, *P*>0.05 in males and *r*=0.09, *P*>0.05 in females) in the right coronary artery lesions groups.

**Table 3:** The CS flow (ml/min) in PCA, the LCA and RCA in non-hypertensive patients with CAD

	Male	Female
PCA	109.6±84.76*	97.04±50.32*
LCA	263.6±94.33*	176±83.65*▵
RCA	310.77±104.33**	207±109.78**▵

* *P* <0.05

** *P* <0.01, unpaired *t* test, compared to the values of PCA of the same gender

▵ *P* <0.05, unpaired *t* test, compared to the values of the males of the same group. CS = coronary sinus; PCA = proximity of coronary artery lesions; LCA = left coronary artery lesions; RCA = right coronary artery lesions; CAD = coronary artery disease.

### ROC analysis

**Fig. 2:**
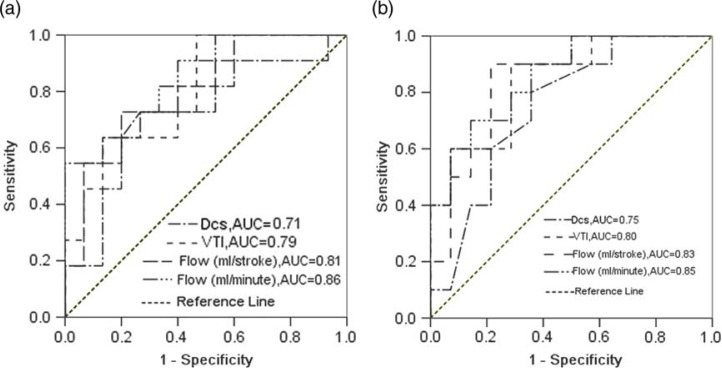
showing the performance of the DCS, the VTI and the CS flow in discrimination between non-patients with CAD or not. **Not clear** (a) males; (b) females. CS = coronary sinus; VTI = velocity time integral; DCS = diameter of coronary sinus; CAD = coronary artery disease; ROC = receiver operating characteristic curve.

With the use of the diameter of CS and blood flow parameters in the CS as the criteria to distinguish patients with CAD from normal subjects, the area under the ROC (AUC) was 0.71 for the diameter of CS, 0.79 for VTI, 0.81 for the CS flow (ml/stroke) and 0.86 for the flow (ml/min) in males; the AUC was 0.75 for the diameter of CS, 0.80 for VTI, 0.83 for the CS flow (ml/stroke) and 0.85 for the flow (ml/min) in females. The AUC for the flow (ml/min) both in males and females was maximal, and the AUC for the VTI and the flow (ml/stroke) were slightly lower, but they were all higher than the AUC for the diameter of CS (*P*<0.05, Fig. 2).

### Discriminant analysis

On the basis of the data depicted above, a discriminant analysis of the cutoff value of the CS flow for predicting a significant coronary artery stenosis (>70%) was conducted. In males, the diagnostic sensitivity, specificity, positive predictive value, negative predictive value, and accuracy of the cutoff value (206 ml/min) of the CS flow were 55/60 (91.67%), 65/80 (81.25%), 55/70 (78.57%), 65/70 (92.86%) and 120/140 (85.71%), respectively, and those of the cutoff value (195 ml/min) of the CS flow in females were 54/63 (85.71%), 54/72 (75%), 54/72 (75%), 54/63 (85.71%), and 108/135 (80%), respectively. All the diagnostic indexes were slightly higher in males than in females (*P*<0.05; Table 4).

**Table 4:** Results of discriminant analysis of the best cutoff values of the CS flow (ml/min) for predicting a significant coronary artery stenosis (>70%) in non-hypertensive patients with CAD

	Flow (ml/min)	
	
Variables	Male	Female
Cutoff value	206	195
*P*-value	<0.001	<0.001
Sensitivity (%)	55/60 (91.67%)	54/63 (85.71%)
Specificity (%)	65/80 (81.25%)	54/72 (75%)
PPV (%)	55/70 (78.57%)	54/72 (75%)
NPV (%)	65/70 (92.86%)	54/63 (85.71%)
Accuracy (%)	120/140 (85.71%)	108/135 (80%)

CAD = coronary artery disease; PPV = positive predictive value; NPV = negative predictive value.

### Reproducibility

Intraobserver and interobserver variability for DCS and VTI ranged from 2.7 to 5.3%. Intraobserver and interobserver variability for blood flow per stroke were 6.5%±2.1% and 6.9%±3.2%, respectively. Intraobserver and interobserver variability for blood flow per min were 7.3%±2.6% and 7.9%±2.4%, respectively.

## Discussion

The results presented here indicate that TTE can effectively detect hemodynamically significant stenosis of the coronary artery in non-hypertensive patients. The reduced antegrade flow in the CS is a sensitive and specific predictor of coronary artery stenosis in males and females at different cutoff values.

The degree of coronary artery stenosis is an important index in the diagnosis and treatment of CAD. Although computed tomography angiography and digital subtraction angiography have been routinely applied to assess coronary artery stenosis, they are not always objective and accurate (11–14). In the clinic, we met several patients who suffered from conspicuous chest pain and had significantly lower blood flow in the CS detected by TTE, but computed tomography angiography and digital subtraction angiography only found a minor coronary artery stenosis (<40%) and electrocardiography (ECG) only showed non-specific changes of ST-T wave. A few days later, some of these patients suffered from myocardial infarction. These events remind us that prompt intervention should be taken into consideration when finding a significantly lower blood flow in the CS.

In routine echocardiographic examinations, we have found that CS can be adequately visualized from the parasternal right ventricular inflow tract view through the approach of TTE in almost all subjects. The flow of CS in this view can be monitored by TTE within a distance of 1–5 cm from the ostium with a <30° Doppler angle between the ultrasound beam and the vessel, which is similar to the same measurement obtained with transesophageal Doppler echocardiography, which ensures a precise measurement of the flow of CS. Therefore, we think that TTE is a highly accurate way of measuring coronary flow velocity, and can be used for evaluating various clinical problems.

Our findings show that normal males had significantly higher coronary blood flow in the CS compared with those of females. This may be due to greater myocardium demands and metabolism in males. In the situation of coronary artery stenosis, the CS flow both in males and females significantly decreased, but the average CS flow in males was also higher than that of females. In addition, we analyzed the impact of PCA, the left and right coronary artery lesions on the CS flow in males and females. The CS flow was significantly less in the PCA group and the left coronary artery lesions group than in the right coronary artery lesions group, and it significantly correlated with the mean percent diameter stenosis, but *not in* the right coronary artery lesions group. These results can be explained by the anatomy of the CS, which is the major venous collection system for the coronaries, especially for the left coronary artery, and the PCA group and the left coronary artery lesions have more impact on the CS flow.

We also compared the performance of the diameter of CS and blood flow parameters (VTI, flow per stroke and flow per min) in the CS in discriminating between patients with or without CAD according to the AUC of the ROC. Flow (ml/min) was finally determined. By discriminant analysis, we obtained the best cutoff values of flow (ml/min) for predicting a significant left coronary artery stenosis (>70%), that is, 206 ml/min for males and 195 ml/min for females. These data suggest that TTE can be a valuable clinical tool in the diagnosis of non-hypertensive patients with CAD by detecting the changes of blood flow in the CS alone (not CFR). Different diagnostic values for males and females should be chosen when the using the antegrade flow in the CS as an indicator of CAD in non-hypertensive patients.

Our study had some limitations. First, the number of participants was limited. New data need to be collected in subsequent studies. Second, the CS flow measurement was sometimes difficult because of interference from heart movements. Even so, TTE, being rapid, reliable, inexpensive, and non-invasive in the assessment of the antegrade flow in the CS, has potential for clinical application. In further studies, all patients (including asymptomatic ones) should undergo assessment of flow in the CS. For patients with abnormal coronary flow, coronary angiography should be performed.

## Conclusions

In non-hypertensive patients, detection of reduced antegrade flow in the CS by transthoracic echocardiography is associated with significant coronary artery stenosis. Males and females have different diagnostic cutoff values. This method could have considerable clinical promise in the diagnosis of CAD.
